# Prevalence of maxillary midline diastema and its societal perception in patients at Remera health center, Rwanda

**DOI:** 10.6026/973206300211567

**Published:** 2025-06-30

**Authors:** Dona Soman, Ramnath Elangovan, Dinesh C. Maganti, Kamikazi Rachel, Muhoza Cedric, Igirubuntu John Peter

**Affiliations:** 1 Department of Prosthetic and Restorative Dentistry, School of Dentistry, University of Rwanda; 2 Department of Periodontics and Community Dentistry, School of Dentistry, University of Rwanda, Kigali, Rwanda; 3 Department of Dental Surgery, School of Dentistry, University of Rwanda, Kigali, Rwanda

**Keywords:** Maxillary midline diastema, perception, society

## Abstract

The prevalence of maxillary midline diastema and its societal perception among patients attending Remera health center, Rwanda is of interest. This descriptive cross-sectional quantitative study was done among patients attending Remera health center. The data collected on the prevalence of midline diastema and its societal perception was measured through SPSS software version 20 by applying descriptive analysis. To analyze the relationship between the variables, inferential statistics were adopted to analyze the relationship between variables. The study investigated the prevalence and perception of maxillary midline diastema, where 27.3% have maxillary midline diastema and 77.3% of all the patients attending Remera health center perceived maxillary midline diastema as a sign of beauty.

## Background:

The prevalence of individuals with diastema from Late Antique and Early Medieval period was approximately 5% and nowadays it is between 3.7% to as much as 36.8% [1]. Israa *et al.* in 2016 stated that it is generally known that the black population (5.5%) has a higher incidence of maxillary midline diastema than the white (3.4%) and Chinese populations (1.7%) [2]. Despite having the high prevalence in black population, it has been scientifically proven that diastema could run between families, and the white population is the most to inherit it. In 2013, Mehdi showed that the percentage of median diastema 12.59% in Pakistan is significantly higher than the prevalence in the United Kingdom 3.4% Caucasians and 1.6% of South Indians [3]. A study among Turkish population showed that midline diastema was observed in 4.5% of the patients and Kuwait to be 26.8% [3]. In 2008, Maria *et al.* declared that the presence of midline diastema was observed in 14.8% of the school children in Ceara, Brazil [4]. Outside of the West world and the Middle East, there is noticeably little literature in this field. But Renata Chalas (2008) demonstrated that the incidence of diastema in African Americans was 16.2%, among Mexican Americans it was 6.6% and in Nigeria the incidence of diastema was 36.8% [1]. The prevalence of midline diastema in Sudanese university students was recorded to be 7.3% [1]. The study by Athumani and Mugonzibwa (2006) showed higher incidence in the Tanzanian population (26%) [3]. The actual value of attraction, which gives a person a perceptual sense of pleasure and fulfillment, is beauty. Everyone is entitled to their own view regarding aesthetics and beauty. Likewise, humans exhibit a variety of attitudes and perception with regard to maxillary midline diastema. In 2022, NK Onyejaka *et al.* published that perception of midline diastema varies among individuals and culture and from professionals and non-professionals. In Nepal, it has been shown that, midline diastema can lead to low self-esteem of an individual. An earlier study in Finland revealed that people with midline diastema are seen to be less intelligent and of low social status. However, in Nigeria, maxillary midline diastema is seen as a sign of beauty hence patients show determination and desperation in seeking its creation [5]. The South Sudanese tribe of Toposa has some intriguing ritualistic practices. From an early age, diastema was intentionally made by placing wooden sticks between the middle maintaining the incisors until adulthood, achieving this marker of mutual formosity. However, the majority of the dentists did not support the artificial creation of midline diastema [6]. Therefore, it is of interest to assess the prevalence of maxillary midline diastema and its societal perception in patients attending Remera Health Center.

## Methodology:

This section elaborates the approach we have used to carry out the research. It includes study area and study population, techniques and procedures which were used to gather information, data processing and analysis.

## Study area:

This research project was conducted in Kigali city, Gasabo district at Remera Heath Center.

## Study design:

The research project was a descriptive cross-sectional study. Data was collected from participants using pointed semi-structured questionnaires; each patient attending Remera health center that was willing to participate was given a questionnaire to fill and the study was carried out between October to January 2024.

## Study population:

The study population for this research project were patients above 18 years of age who were attending REMERA Health Center and willing to consent and participate in this study within the six-week time period.

## Inclusion criteria:

The following inclusion criteria were used to select population and sample:

[1] Adult patients above 18 years

[2] All patients attending Remera Health Center were willing to participate and signed informed consent.

## Exclusion criteria:

## The exclusion criteria were:

[1] Individual with any missing permanent maxillary central incisor.

[2] All other individuals who are not attending Remera health center and who do not consent to participate were excluded.

## Sample size:

The sample size to be used was calculated using the total average number of patients attending Remera health center in six weeks at the time of data collection. The sample size estimated is 384 in total number.

## Data collection methods and strategy:

The data for this study was collected by using semi-structured questionnaires. A structured questionnaire was administered to participants that includes a set of color smile photographs with varying sizes of maxillary midline diastema (narrowest = 0 mm; widest = 4 mm). Subjects were required to rate the different color photographs using a rating scale, where 5 = very attractive; 4 = attractive; 3 = acceptable; 2 = unattractive; 1 = very unattractive. The codes collapsed by combining codes 1/2, and codes 4/5 to give three categories: attractive, acceptable, and unattractive. Every participant was given a questionnaire to fill in and after filling in the copies was collected to be analyzed. The process of data collection started after permission was granted by IRB-UR CMHS and Remera health center.

## Independent variables:

The independent variables in this study are comprised of the following characteristics Age, Gender, Level of social economic status, Level of education, Profession of the participants attending Remera health center.

## Dependent variables:

The dependent variables of this study included smile attractiveness and prevalence. These measures were assessed by using LIKERT SCALE-formulated questionnaire.

## Data analysis:

The data collected on the prevalence of midline diastema and its societal perception was measured through SPSS software version 25 by applying descriptive analysis and was used to measure the frequency. To analyze the relationship between the variables, inferential statistics were adopted to analyze the association between variables.

## Ethical consideration:

Participation in the study was only upon the willingness of the participants and individual confidentiality was ensured as well as respect to the study participants. It is in this context that the requirement to sign consent by each participant was upheld before participating in the study. Research proposal was submitted to the ethical committee of the college of medicine and health science to be reviewed and approved with ethical clearance before the initiation of research process.

## Results:

The study consisted of 384 participants, with 234 (60.9%) females and 65 (39.1%) males (Table 1 - see PDF). The age range data indicates that most patients fall within the age groups of 18-30 and 31-40, comprising 34.1% and 26.8% of the total sample, respectively, with relatively lower proportions in older age categories. A significant portion, constituting 44.0%, identified in Ubudehe category 1, and 33.6% in Ubudehe category 2, 17.4% in Ubudehe category 3, while 4.4% stated were found to be in Ubudehe Category 4. 40.4% of participants are illiterate and 33.6% completed primary education. 20.1% had attained secondary education. 28.3% had completed primary education, while 6.0% had finished university education. Majority of participants fall in the Non-dental Inclined range (97.7%), leaving 2.3% to be in the Dental Inclined range. In this study, the prevalence in males is (26.7%) and females (27.8%) while in the age groups 18-30 exhibit the highest prevalence (48.1%) whereas the above 60 years has the least (5.4%) (Table 2 - see PDF). The ubudehe category 4 (58.8%) had the highest prevalence than the ubudehe category 1 (14.2%) whereas the tertiary level of education (78.3%) reported with the significant portion than the no basic level of education (14.8%). Also, participants who were dental inclined (66.7%) had the highest prevalence than the non-dental inclined (26.4%). In this study, the majority of 18-30 years' age group (77.1%) perceived maxillary midline diastema as a sign of beauty. The results showed that 64.0% male and 85.9% female perceived a maxillary midline diastema as a sign of beauty (Table 3 - see PDF). With most of the Ubudehe category I (85.2%) and an illiterate background (86.5%) considered maxillary mid-line diastema a sign of beauty. Both dental (7%) and non-dental (77.3%) inclined had profound consideration of maxillary midline diastema. The 0mm diastema was perceived as attractive by a greater proportion of both males (86.7%) and females (77.8%) as well as most participants in all the age groups with the highest proportion in age group 51-60 years (86.9%) (P= 0.031) (Table 4 - see PDF). Also, most of the subjects in all the Ubudehe category, educational levels and both dental and non-dental inclined graded 0mm Maxillary Midline diastema as attractive. Majority of the participants still perceived 0-1MM maxillary midline diastema as attractive although the percentages raised higher in females (91.1%) than males (72.7%) (Table 5 - see PDF). The highest proportion that found the maxillary midline diastema attractive was among the 41-50 years (92.3%), Ubudehe category 1 (91.1%) and Illiterate background (91.0%). Both professional dental related and non-dental related still found 0-1MM maxillary diastema as attractive. The proportion of participants who considered the attractiveness of 1-2MM maxillary midline diastema reduced further. (47.3%) males and (66.1%) females perceived the 1-2MM maxillary midline diastema as acceptable. The highest proportion who considered it acceptable was found among the age group 31-40 years (65.0%) (P= 0.33), Ubudehe category 2 (66.4%), participants with primary level of education (67.4%) (P<0.01) and among the professionally dental inclined (22.2%) (Table 6 - see PDF). The 2-4MM diastema was perceived as unattractive by a greater proportion of both males (91.9%) and females (88.0%). A greater percentage of participants in all age groups, all as well as participants with tertiary level of education and also all dental and non-dental inclined professions perceived the 2-4MM maxillary midline diastema as unattractive (Table 7 - see PDF). In this study, the desire to remove the maxillary midline diastema was great in males (18.7%) than in females (6.0%) (Table 8 - see PDF). The dental inclined (22.2%) had a more significant proportion than non-dental inclined (10.7%). The tertiary level of education participants (26.1%) has a great percentage in desire to remove the maxillary midline diastema as well as the participants who were in Ubudehe category 4 (25.4%). In this study, females (32.1%) had a greater percentage for the desire to create the maxillary midline diastema than males (5.3%) whereas the non-dental inclined (21.9%) had most significant proportion to create the maxillary midline diastema (Table 9 - see PDF).

## Discussion:

In this study, the prevalence and perception of the maxillary midline diastema is in relation to different socio-demographic characteristics including age, gender, ubudehe category, level of education and profession. There is a varied pattern in the prevalence of maxillary midline diastema where the highest presence was in the younger age (18-30 years) than the older age as noticed in other many studies worldwide. Females had the higher prevalence than males. In regard to the socioeconomic background, ubudehe category 4 had the greatest prevalence than any other category. The university educational background and non-dental inclined profession had the most prevalence in comparison to all other categories respectively. In general, women are more concerned about their appearance than men do. In this study, there was a slight difference between how the male and female participants perceived midline diastema, with most females preferring diastema than the males. This result contrasts with another Baghdad study that found a greater preference for midline diastema among men. According to this study, participants with illiterate and primary education levels thought midline diastema was more attractive than those with higher education levels. Higher educated subjects are less likely to find a midline diastema attractive. This conclusion can be explained by the fact that participants with greater educational backgrounds tend to be better informed and have been exposed to more information sources, which results in more informative views and perceptions. In this study, diastema diameters between 0-1 mm were deemed attractive by the majority of respondents. An additional increase in diameter produced a decline in the respondents' preference for it. According to a prior study in Nigeria, the majority of respondents from Southwest Nigeria (72.8%) thought that having a diastema was a sign of beauty. However, the respondents did not seem to value a gap larger than 4 mm in diameter, and this was statistically correlated with respondents' age and level of education. A previous study conducted with Jordanians found that midline diastema was not thought to be attractive.

In this study involving dental and non-dental inclined, it was shown that dental inclined considered a smile to be ugly if the midline diastema width was greater than 2mm. A diastema larger than 2mm was typically not seen as an asset of beauty, even if the study's participants thought that a midline diastema was an indication of beauty. According to the study's findings, the age group older than 40 years old had the highest percentage of participants who thought the midline diastema was appealing as its width expanded from 0-1 mm. The attractiveness of the diastema decreased overall when its width increased 2-4 mm, with the age group over 40 years old still having the largest percentage of subjects pertaining to the diastema at such width. Only forty-two (10.9%) of 384 participants would like to close midline diastema when it exists. Out of this, 42.8% of them were in the 18-30 years age group and this was statistically significant (p=0.002) [7]. Also, more males (18.7%) than females (1.8%) would like to close midline diastema and this was statistically significant (P=0.001). However, 21.6% (83/384) of all study participants wished to create the maxillary midline diastema. A prior study in southeastern Nigeria showed that 34% of patients who attended a dental clinic had midline diastema artificially created to enhance their beauty. However, in this about (18.7%) of all the respondents and significantly males reported that they will seek treatment to close the midline diastema assuming they have it. This is a pointer to the fact that males in this study population were not fascinated by midline diastema when compared to females.

## Conclusion:

A study on maxillary midline diastema (MMD) revealed that 27.3% of participants had MMD, with 77.3% perceiving it as a sign of beauty. Gaps of 0-1 mm were considered attractive, while those measuring 2-4 mm were deemed unattractive. Females (32.1%) were more inclined than males (5.3%) to express interest in artificially creating MMD. Factors such as age, gender, socioeconomic status, education, and dental knowledge significantly influenced perceptions of MMD. The study underscores the psychological and social impacts of MMD, including low self-esteem in individuals with wider gaps, and highlights the need for oral health education, public health campaigns, and personalized treatment approaches to enhance the quality of life and mental well-being of those affected.
